# *UGT1A1* Gene Polymorphism Contributes as a Risk Factor for Lung Cancer: A Pilot Study with Patients from the Amazon

**DOI:** 10.3390/genes13030493

**Published:** 2022-03-11

**Authors:** Esdras E. B. Pereira, Luciana P. C. Leitão, Roberta B. Andrade, Antônio A. C. Modesto, Bruno M. Fernandes, Rommel M. R. Burbano, Paulo P. Assumpção, Marianne R. Fernandes, João F. Guerreiro, Sidney E. B. dos Santos, Ney P. C. dos Santos

**Affiliations:** 1 Laboratory of Human and Medical Genetics, Institute of Biological Science, Federal University of Pará, Belem 66077-830, Brazil; esdrasedgarbp@gmail.com (E.E.B.P.); robertaborgesandrade@gmail.com (R.B.A.); antonioacm@ufpa.br (A.A.C.M.); rommelburbano@gmail.com (R.M.R.B.); joaofg@ufpa.br (J.F.G.); sidneysantos@ufpa.br (S.E.B.d.S.); npcsantos.ufpa@gmail.com (N.P.C.d.S.); 2 Oncology Research Center, Federal University of Pará, Belem 66073-005, Brazil; colaresluciana@gmail.com (L.P.C.L.); brunomelllo1@gmail.com (B.M.F.); assumpcaopp@gmail.com (P.P.A.)

**Keywords:** genetic polymorphism, *UGT1A1*, biomarker, lung cancer, smoking

## Abstract

Lung cancer is one of the most frequent neoplasms in the world. Because it is a complex disease, its formation occurs in several stages, stemming from interactions between environmental risk factors, such as smoking, and individual genetic susceptibility. Our objective was to investigate associations between a *UGT1A1* gene polymorphism (rs8175347) and lung cancer risk in an Amazonian population. This is a pilot study, case-controlled study, which included 276 individuals with cancer and without cancer. The samples were analyzed for polymorphisms of the *UGT1A1* gene (rs8175347) and genotyped in PCR, followed by fragment analysis in which we applied a previously developed set of informative ancestral markers. We used logistic regression to identify differences in allelic and genotypic frequencies between individuals. Individuals with the TA7 allele have an increased chance of developing lung adenocarcinoma (*p* = 0.035; OR: 2.57), as well as those with related genotypes of reduced or low enzymatic activity: TA6/7, TA5/7, and TA7/7 (*p* = 0.048; OR: 8.41). Individuals with homozygous TA7/7 have an increased chance of developing squamous cell carcinoma of the lung (*p* = 0.015; OR: 4.08). Polymorphism in the *UGT1A1* gene (rs8175347) may contribute as a risk factor for adenocarcinoma and lung squamous cell carcinoma in the population of the Amazon region.

## 1. Introduction

Lung neoplasms are among the most prevalent types of cancer in the world population, representing 11.4% of all registered cancers, and are responsible for 18% of cancer deaths [1]. This frequency varies between countries, depending on their demographic characteristics, smoking rate, and level of economic development [2]. Lung cancer can be divided into two categories, small cell lung cancer (SCLC), responsible for 15–20% of cases; and non-small cell lung cancer (NSCLC), which represents 80–85% of cases [3,4].

The well-known risk factor for the development of lung cancer is smoking, given the presence of substances contained in tobacco associated with carcinogenesis [5]. However, studies have shown that about half of new cases are in people who have never smoked, or have stopped smoking for many years [6]. This shows that pulmonary carcinogenesis is a complex and gradual process, with complex interactions between environmental risk factors and individual genetic susceptibility [7].

Genetic susceptibility to lung cancer, often modulated by smoking behavior, has been investigated [8]. Disease risk may be associated with genetic variables such as single nucleotide polymorphisms (SNPs) in genes related to metabolism, DNA damage repair, and cell cycle control [9,10]. This knowledge is important when it comes to lung cancer, and its use can favor the development of population screening tools [11].

The *UGT1A1* gene originates from the UDP-glucuronosyltransferase enzyme, which is active in the glucuronidation metabolic pathway, one of the main biotransformation pathways of xenobiotics [12,13]. This gene has been linked to the development of several types of cancer: colon, breast, and prostate. The UGT1A1 enzyme plays an important role in the detoxification and metabolization of several carcinogens [14,15].

The aim of this study was to investigate possible associations between *UGT1A1* gene polymorphism (rs8175347) and lung cancer risk in an Amazonian population.

## 2. Materials and Methods

### 2.1. Ethical Conformity

This pilot study was approved by the Research Ethics Committees of the Oncology Research Center, under protocol 927.808/2014, and by the João de Barros Barreto University Hospital, under protocol number 941.207/2015, both in the city of Belém-Pará, Amazon region of Brazil. All participants signed an informed consent form.

### 2.2. Case and Control

Participants were recruited from public healthcare centers, of both sexes, not belonging to the same family nucleus, and of the same socioeconomic level. Data and samples were collected from 276 individuals, of which 80 were patients with primary lung cancer (case group), defined and classified in the histopathological examination, and 196 patients were without any type of cancer (control group). Both groups had a survey of demographic and clinical data, which included age, sex, and smoking.

### 2.3. DNA Extraction and Quantification

Extraction of genomic DNA from peripheral blood leukocytes was conducted using a Mini Spin Plus Kit (P. 250, Biopur, Biometrix) according to the manufacturer’s recommendations. DNA concentration and purity were measured with a NanoDrop 1000 spectrophotometer (Thermo Scientific NanoDrop 1000; NanoDrop Technologies, Wilmington, DE, USA).

### 2.4. Genotyping

The *UGT1A1* gene polymorphism (rs8175347) was genotyped by a multiplex PCR reaction followed by capillary electrophoresis. For the amplifications, primers were used: F:5′-CTCTGAAAGTGAACTCCCTGCT-3′; R:5′-AGAGGTTCGCCCTTCCTAT-3′. Analysis of the PCR amplicons was performed from an electrophoresis using the ABI Prism 3130 sequencer and GeneMapper ID v.3.2 software [16]. The genotypes found were: TA6/6 (UGT1A1*1/*1), TA6/7 (UGT1A1*1/*28), T5/7 (UGT1A1*36/*28) and TA7/7 (UGT1A1*28/*28). In the literature, these genotypes can be grouped by the degree of enzymatic activity of UGT1A1 as: normal (TA6/6), reduced (TA6/7 and TA5/7), and low (TA7/7) enzymatic activity [17].

### 2.5. Hardy–Weinberg Equilibrium Analysis (HWE)

The allelic and genotypic frequency of the polymorphism was determined by direct counting of alleles, and then Hardy–Weinberg equilibrium (HWE) was calculated using the default parameters of the Arlequin 3.5.1.2 software (Swiss Institute of Bioinformatics, Bern, Switzerland). 

### 2.6. Genetic Ancestrality Analysis

Genotyping was performed to analyze the ancestry of the samples, performed according to Ramos et al. [18], using 61 informative markers of autosomal ancestry in three multiplex PCR reactions. Amplicons were analyzed by electrophoresis using the ABI Prism 3130 sequencer and GeneMapper ID v. 3.2 software (Applied Biosystems, Life Technologies, Carlsbad, CA, USA). The individual proportions of European, African, and Amerindian genetic ancestry were estimated using Structure v. 2.3.3 software (Stanford University, Stanford, CA, USA), assuming three parental populations [19].

### 2.7. Statistical Analysis

All statistical analyzes were performed using the SPSS 20.0 software (IBM, Armonk, NY, USA) statistical package. For comparative analysis between the study groups with regard to demographic and clinical variables, Pearson’s Chi-square and the Mann–Whitney test were applied. To analyze the association of polymorphisms with risk to lung cancer and the histological types, a logistic regression was performed, estimating the odds ratios (OR) and their 95% confidence intervals (CI). The variables of sex, age, and smoking were controlled in this multivariate analysis, to avoid a confounding association. A significance level of *p* < 0.05 was considered for all statistical analyses.

## 3. Results

In the results of demographic and clinical analyses, it can be observed that the groups differed in terms of sex, age, and smoking. Most cases of lung cancer were squamous cell carcinoma, followed by large cell carcinoma and adenocarcinoma (Table 1). The ancestry analyses performed revealed that the case and control groups had a similar ancestral genomic profile, with a greater European contribution to both populations (Table 1).

It was evidenced that the *UGT1A1* gene polymorphism (rs8175347) was present in the HWE (*p* = 0.340). Four genotypes were presented, TA6/6, TA6/7, TA5/7, and TA7/7, with frequencies of 41%, 45%, 1%, and 13% respectively. As for the allele frequency, three alleles were observed, TA5, TA6, and TA7, with 0.5%, 63.4%, and 36.1%, respectively.

The allele frequencies between the control and lung cancer groups and their histological types are listed in Table 2. It was observed that the TA7 allele was present more frequently in the lung adenocarcinoma group (54.1%). In the analysis for lung adenocarcinoma, it was observed that individuals with the TA7 allele were about 2.5 times more likely to develop the disease (*p* = 0.035; OR: 2.57; 95% CI: 1.07–6.20).

The genotypic frequencies between the control and lung cancer groups and their histological types are listed in Table 3. The TA7/7 genotype was more frequent in the squamous cell carcinoma group (22.0%). Individuals with the TA7/7 genotype were about four times more likely to develop lung squamous cell carcinoma (*p* = 0.015; OR: 4.08; 95% CI: 1.32–12.61).

The genotypic frequencies by the degree of UGT1A1 enzymatic activities, between the control and lung cancer groups and their histological types, are listed in Table 4. Genotypes with reduced or low enzyme activity (TA6/7, TA5/7, TA7/7) were more frequent in the lung adenocarcinoma group (91.7%). Furthermore, individuals with a low or reduced degree of enzyme activity genotypes were about 8 times more likely to develop the disease (*p* = 0.048; OR: 8.41; 95%CI: 1.02–69.55).

## 4. Discussion

In the world, lung neoplasms are the second most common cancer diagnosis in both sexes, being more frequent among men with an average age of 70 years, and associated with tobacco consumption [1,20,21]. In Brazil, lung cancer is the third most common cancer among men and the fourth most common cancer among women, and 85% of diagnosed cases are associated with the consumption of tobacco derivatives [22,23]. This is similar to what was observed in our study, where the group of individuals with lung cancer was mainly composed of men aged over 65 years and with a history of smoking.

The prevalence of lung cancer in men is often associated with smoking, as the proportion of men who smoke is higher than that of women. In addition, men are also exposed to carcinogens in some occupational activities, which contributes to the frequency being higher in this group [24,25,26]. However, these gender differences vary between developed and developing countries, due to differences in tobacco consumption, and exposure to exogenous and endogenous risk factors [1,5].

Regarding age, it is observed that about 34% of lung cancer cases in the world are diagnosed between 65 and 74 years of age, and the elderly population comprises 62% of all cases [1]. Aging favors genomic instability, leading to the accumulation of cells with different molecular aberrations that alter internal homeostasis, increasing susceptibility to carcinogens and, consequently, lung carcinogenesis [27]. According to Schneider et al. (2021) some histological types of lung cancer are caused by the expression of oncogenic drivers, which can be stratified by age, such as adenocarcinoma, revealing the interaction between aging and lung carcinogenesis [28].

In our study, the most prevalent histological type was squamous cell carcinoma (51.7%), followed by large cell carcinoma (17.5%), and adenocarcinoma (15.0%). In Brazil, 86.7% of cases are NSCLC and 13.7% SCLC. Among NSCLC, the most common histological type was adenocarcinoma (50.0%), followed by squamous cell carcinoma (42.1%), and large cell carcinoma (7.9%) [29]. these differences may reflect the reduction in cases of squamous cell carcinoma and the increase in adenocarcinoma in the last 30 years, due to changes in the pattern of tobacco consumption in some regions of the country [30].

Smoking is the well-known risk factor for developing lung cancer [5,6]. Pulmonary carcinogenesis is a complex process, with interactions that modulate the potential risks for the disease [7,31]. Many studies have described how germline variants influence susceptibility to lung cancer, including those linked to smoking [32,33,34].

In the present study, the analysis of the *UGT1A1* gene polymorphism (rs8175347) showed relevant results. The TA7/7 genotype was present in 13% of the investigated individuals. In Brazil, the frequency of the TA7/7 genotype of this polymorphism occurs in between 3 and 17% of the population, being more frequent among Afrodescendants and less frequent among Amerindians [35].

As for allelic frequency, the TA7 allele was frequent in 36.1% of the individuals studied. This frequency is similar to that observed in the wider world [36], where the distribution of the TA7 allele is 34.9%, in Latin America 31.4%, and in Brazil between 30.0 and 33.0% [37,38,39]. Studies show that the TA7 allele has a frequency of 42–56% in Afrodescendants, 26–31% in Caucasians, and only 9–16% in Asian populations [40].

In our study, individuals with genotypes related to reduced or low enzymatic activity (TA6/7, TA5/7 and TA7/7) had increased chances (*p* = 0.048; OR: 8.41, 95%CI: 1.07–69.55) for the development of adenocarcinoma of lung. Similar results were observed in the allelic frequency: individuals with the TA7 allele have an increased chance of developing lung adenocarcinoma (*p* = 0.035; OR: 2.57; 95%CI: 1.07–6.20). Furthermore, individuals with the TA7/TA7 genotype have an increased chance of developing squamous cell carcinoma of the lung (*p* = 0.015; OR:4.077; 95%CI:1.318–12.612).

The variant allelic TA7 is characterized by seven thymine-adenine (TA) repeats within the promoter region, unlike the wild-type TA6 allele which has six TA repeats. This extra repeat impairs proper gene transcription [41,42,43]. This results in a 25–70% reduction in enzyme activity, depending on the presence of one or two TA7 variant alleles, respectively, which reduce glucuronidation [44,45,46].

UGT1A1 enzymes were observed in the glucuronidation of estradiol and of a precursor of the potent organic carcinogen benzo(α)pyrene-7,8-dihydrodiol-9,10-epoxide (BPDE), which is found in cigarette smoke [40]. There is a correlation between *UGT1A1* genotypes with the expression of UDP-glucuronosyltransferase and glucuronidation activity. Individuals with genotype TA7/7 have a lower level of precursor carcinogen in liv-er microsomes when compared to those with genotypes TA6/7 or TA6/6 [40].

In the present study, smoking was present in 83.7% of patients with lung cancer, similar to that observed in other surveys, where between 80% and 90% of lung cancers are attributed to smoking [47,48]. Tobacco contains dozens of carcinogenic agents that are harmful to humans, who undergo biotransformation through various metabolic path-ways [49,50]. Studies show that genetic polymorphisms in genes that encode enzymes involved in the metabolism of tobacco carcinogens, such as UGT1A1, can affect the individual risk of lung cancer [51,52].

The reduction in or low enzymatic activity of UGT1A1 reduces glucuronidation, increasing exposure to the carcinogens present in tobacco, which favor carcinogenesis [42]. Our study observed this association. Individuals with the genotypes of reduced or low enzymatic activity, or with an allele TA7 or with genotype homozygous TA7/7, had a higher chance of some histological types of lung cancer. It is possible that these associations were not observed in all histological types due to the small sample size in some clusters in this pilot study.

This finding corroborates what was observed by Nishikawa et al. (2016) [53] in their study of 194 patients with lung cancer, who found that the genotype homozygous TA7/7 increases the risk of developing lung cancer by approximately five times. They observed that squamous cell carcinoma was predominant in the lung cancer patients with genotype homozygous TA7/7 and smoking history, similar to our study.

This reinforces the findings of other studies that indicate that reducing the activity or expression of UGT1A1 can influence the elimination of precursors to carcinogens [14,48]. This relationship facilitates the understanding of the identification of associations between the TA7 allelic variant with the susceptibility to oxidative damage and, consequently, the increased risk for other types of cancer, such as breast, ovarian, prostate, head and neck, and colorectal cancers, reported in some populations [48,54,55,56,57].

In addition to the susceptibility to the development of lung cancer, studies regarding polymorphism in the *UGT1A1* gene (rs8175347) have identified its association with the response to chemotherapy with irinotecan. The reduced enzymatic action of UGT1A1, due to the TA7 variant, may be associated with severe toxicities in patients receiving irinotecan, including patients with lung cancer. This shows this polymorphism is a strong candidate for use in clinical practice as well [57,58,59].

Importantly, this study is one of the few that has investigated the association between how polymorphism that can alter the length of the *UGT1A1* gene promoter and the risk of lung adenocarcinoma and squamous cell carcinoma. Further epidemiological investigations involving larger groups of individuals should be conducted to confirm the results and determine whether the TA7 variant allele is an isolated risk factor or associated with environmental factors such as smoking.

The validation of these findings may favor, in the future, the screening of individuals with greater susceptibility to developing the disease by facilitating the establishment of personalized preventive measures for early diagnosis, consequently reducing the cost for health services and lowering mortality rates from this malignant neoplasm.

## 5. Conclusions

The *UGT1A1* gene polymorphism (rs8175347) showed a significant association with lung squamous cell carcinoma and lung adenocarcinoma in the population of the Amazon region. Individuals with homozygous TA7/7 had an increased risk of lung squamous cell carcinoma. Those with the TA7 allele or with genotypes associated with reduced or low UGT1A1 activity had an increased risk for lung adenocarcinoma.

## Figures and Tables

**Table 1:** Demographic and clinical characteristics of the investigated groups.

Characteristics	Case (*n* = 80)	Control (*n* = 196)	*p*-Value
**Sex**			
Male	56 (70.0%)	62 (31.6%)	<0.001 ^a,^*
Female	24 (30.0%)	134 (68.4%)	
**Age (years)**			
Median (p25–p75%)	62.0 (55.0–69.0)	69.0 (64.0–76.0)	<0.001 ^b,^*
**Smoking**			
Never smoked	13 (16.33%)	99 (50.5%)	<0.001 ^a,^*
Smoker	67 (83.7%)	97 (49.5%)	
**Ancestry**			
European	47.6 ± 18.0	45.2 ± 17.0	0.334 ^b^
Amerindian	30.0 ± 14.1	30.6 ± 14.8	0.888 ^b^
African	22.3 ± 12.4	24.2 ± 13.8	0.313 ^b^
**Histology**			
Squamous Cell Carcinoma	41 (51.3%)	-	NA
Large Cell Carcinoma	14 (17.5%)	-	
Adenocarcinoma	12 (15.0%)	-	
Others	13 (16.2%)	-	

NA, not applicable. ^a^ Chi-square Test; ^b^ Mann–Whitney Test; * *p*-value < 0.05.

**Table 2:** Risk for lung cancer and its histological types, associated with the *UGT1A1* gene polymorphism allele.

Groups	*UGT1A1* (rs8175347) Allelotypes			
	(TA)7	(TA)5 or (TA)6	OR (95% IC)	*p*-Value ^a^
Controls	139 (35.5%)	253 (64.5%)	-	-
Lung Cancer	60 (37.5%)	100 (62.5%)	1.24 (0.80–1.93)	0.331
Squamous Cell Carcinoma	35 (42.7%)	47 (57.3%)	1.56 (0.89–2.73)	0.116
Large Cell Carcinoma	5 (17.9%)	23 (82.1%)	0.43 (0.16–1.18)	0.103
Adenocarcinoma	13 (54.1%)	11 (45.9%)	2.57 (1.07–6.20)	0.035 *
Others	7 (26.9%)	19 (73.1%)	0.76 (0.30–1.95)	0.573

OR, odds ratio; CI, confidence interval; ^a^ Logistic regression adjusted for sex, age, and smoking. * *p*-value < 0.05.

**Table 3:** Risk for lung cancer and its histological types, associated with the *UGT1A1* gene polymorphism genotypes.

Groups	*UGT1A1* (rs8175347) Genotypes			
	TA7/7	TA6/6, TA6/7 or TA5/7	OR (95% IC)	*p*-Value ^a^
Controls	23 (11.7%)	173 (88.3%)	-	-
Lung Cancer	13 (16.3%)	67 (83.7%)	2.04 (0.85–4.92)	0.110
Squamous Cell Carcinoma	9 (22.0%)	32 (78.0%)	4.08 (1.32–12.61)	0.015 *
Large Cell Carcinoma	1 (7.1%)	13 (92.9%)	0.77 (0.09–6.61)	0.815
Adenocarcinoma	2 (16.7%)	10 (83.3%)	3.19 (0.53–19.05)	0.204
Others	1 (7.7%)	12 (92.3%)	0.73 (0.08–6.40)	0.780

OR, odds ratio; CI, confidence interval; ^a^ Logistic regression adjusted for sex, age, and smoking. * *p*-value < 0.05.

**Table 4:** Risk for lung cancer and its histological types, associated with degree of UGT1A1 enzymatic activities.

Groups	*UGT1A1* (rs8175347) Genotypes by Degree of Enzymatic Activity			
	Low or Reduced	Normal	OR (95%IC)	*p*-Value ^a^
Controls	116 (59.2%)	80 (40.8%)	-	-
Lung Cancer	47 (58.8%)	33 (41.2%)	1.09 (0.59–1.99)	0.788
Squamous Cell Carcinoma	26 (63.4%)	15 (36.6%)	1.23 (0.56–2.70)	0.604
Large Cell Carcinoma	4 (28.6%)	10 (71.4%)	0.29 (0.08–0.99)	0.058
Adenocarcinoma	11 (91.7%)	1 (8.3%)	8.41 (1.02–69.55)	0.048 *
Others	6 (46.2%)	7 (53.8%)	0.69 (0.21–2.27)	0.543

OR, odds ratio; CI, confidence interval; ^a^ Logistic regression adjusted for sex, age, and smoking. * *p*-value < 0.05.
